# Supplementary material for: Inhibition of Mitochondrial Antioxidant Defense and CDK4/6 in Mesothelioma
Source: Molecules. 2023 May 27;28(11):4380. doi: 10.3390/molecules28114380 (PMC10254447; doi:10.3390/molecules28114380)
Supplement: Supplementary file 1 [file molecules-28-04380-s001.zip › molecules-2065080-supplementary.pdf]

TREATMENT	UnT		AU 1.25 µM		
Gene	mean	SD	mean	SD	2052 n=3
Cdc2	1	0	0.24	0.09	
CDK2	1	0	0.15	0.02	
CDK4	1	0	1.12	0.33	
CDK6	1	0	0.54	0.03	
Cyclin A	1	0	0.01	0.00	
Cyclin B1	1	0	0.18	0.19	
Cyclin D1	1	0	0.90	0.42	
Cyclin E1	1	0	1.04	0.18	

TREATMENT	UnT		AU 1.25 µM		
Gene	mean	SD	mean	SD	2373 n=2
Cdc2	1	0	1.19	0.29	
CDK2	1	0	0.96	0.46	
CDK4	1	0	0.88	0.63	
CDK6	1	0	0.88	0.97	
Cyclin A	no signal				
Cyclin B1	1	0	0.48	0.45	
Cyclin D1	1	0	0.96	0.06	
Cyclin E1	1	0	1.30	0.34	

TREATMENT	UnT		AU 1.25 µM		AU 2.5 µM	
Gene	mean	SD	mean	SD	mean	SD
Cdc2	1	0	0.24	0.22	0.16	0.18
CDK2	1	0	0.76	0.07	0.50	0.28
CDK4	1	0	0.62	0.25	0.93	0.15
CDK6	1	0	1.09	0.44	1.09	0.28
Cyclin A	1	0	0.39	0.04	0.04	0.05
Cyclin B1	1	0	0.39	0.15	0.13	0.12
Cyclin D1	1	0	0.36	0.29	0.23	0.19
Cyclin E1	1	0	0.82	0.18	0.87	0.25

TREATMENT	UnT		PD 35 µM	
**Gene**	**mean**	**SD**	**mean**	**SD**
**Cdc2**	1	0	0.09	0.10
**CDK2**	1	0	0.76	0.06
**CDK4**	1	0	1.69	0.00
**CDK6**	1	0	0.55	0.20
**Cyclin A**	1	0	0.06	0.01
**Cyclin B1**	1	0	0.06	0.00
**Cyclin D1**	1	0	3.27	1.57
**Cyclin E1**	1	0	1.29	0.20

TREATMENT	UnT		PD 5 µM	
**Gene**	**mean**	**SD**	**mean**	**SD**
**Cdc2**	1	0	0.03	0.05
**CDK2**	1	0	2.55	0.35
**CDK4**	1	0	1.79	0.26
**CDK6**	1	0	1.25	0.25
**Cyclin A**	1	0	0.14	0.02
**Cyclin B1**	1	0	0.14	0.04
**Cyclin D1**	1	0	1.98	0.42
**Cyclin E1**	1	0	2.38	0.56

2452 n=3

TREATMENT	UnT		PD 12.5 µM	
**Gene**	**mean**	**SD**	**mean**	**SD**
**Cdc2**	1	0	0.06	0.01
**CDK2**	1	0	0.97	0.91
**CDK4**	1	0	1.44	0.48
**CDK6**	1	0	1.86	0.56
**Cyclin A**	1	0	0.36	0.61
**Cyclin B1**	1	0	0.36	0.31
**Cyclin D1**	1	0	0.98	1.35
**Cyclin E1**	1	0	1.64	0.40

PD 70 µM

mean	SD	2050 n=2
0.08	0.09	
1.30	0.02	
1.41	0.12	
1.36	0.18	
-0.08	0.11	
0.03	0.04	
2.75	1.22	
1.96	0.01	

PD 10 µM

mean	SD	2373 n=3
0.02	0.02	
1.85	0.55	
1.14	0.30	
1.25	0.06	
0.03	0.03	
0.13	0.15	
2.07	0.38	
2.68	0.41	

PD 25 µM

mean	SD	2452 n=2
0.03	0.03	
0.71	0.30	
0.90	0.03	
1.60	0.39	
0.03	0.04	
0.03	0.04	
0.24	0.03	
2.10	0.41	
